# Supplementary figures and images for: LABRAT reveals association of alternative polyadenylation with transcript localization, RNA binding protein expression, transcription speed, and cancer survival
Source: BMC Genomics. 2021 Jun 26;22:476. doi: 10.1186/s12864-021-07781-1 (PMC8234626; doi:10.1186/s12864-021-07781-1)

A

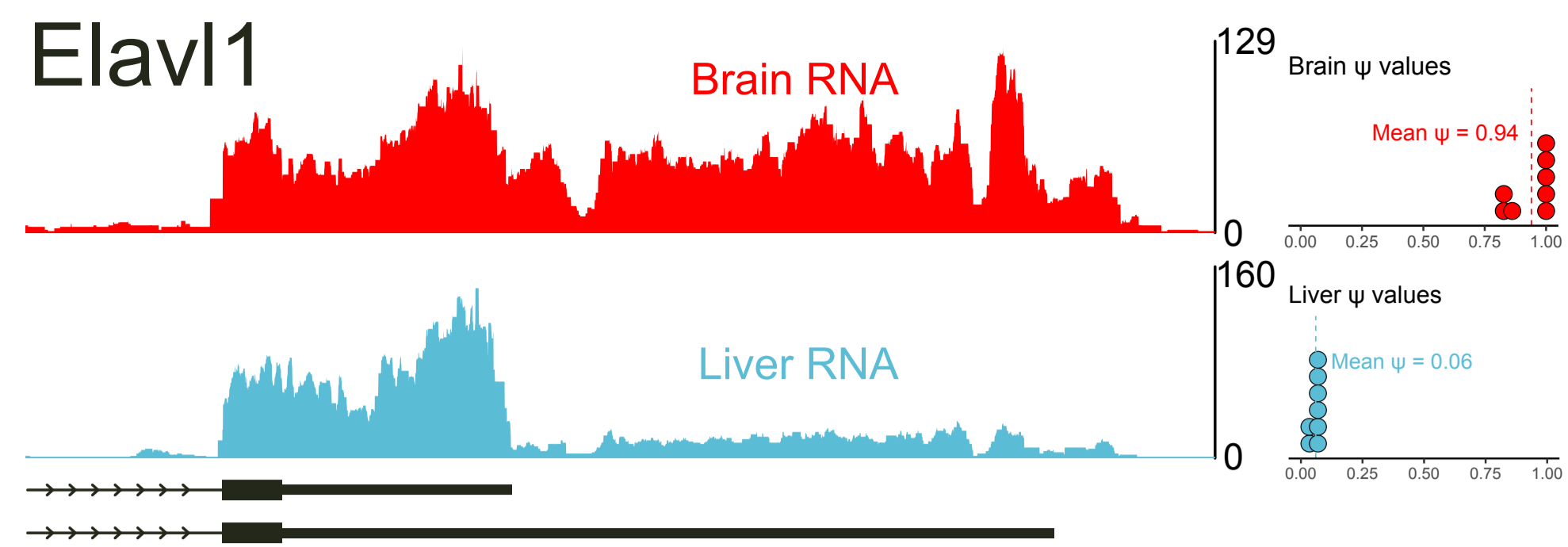

B

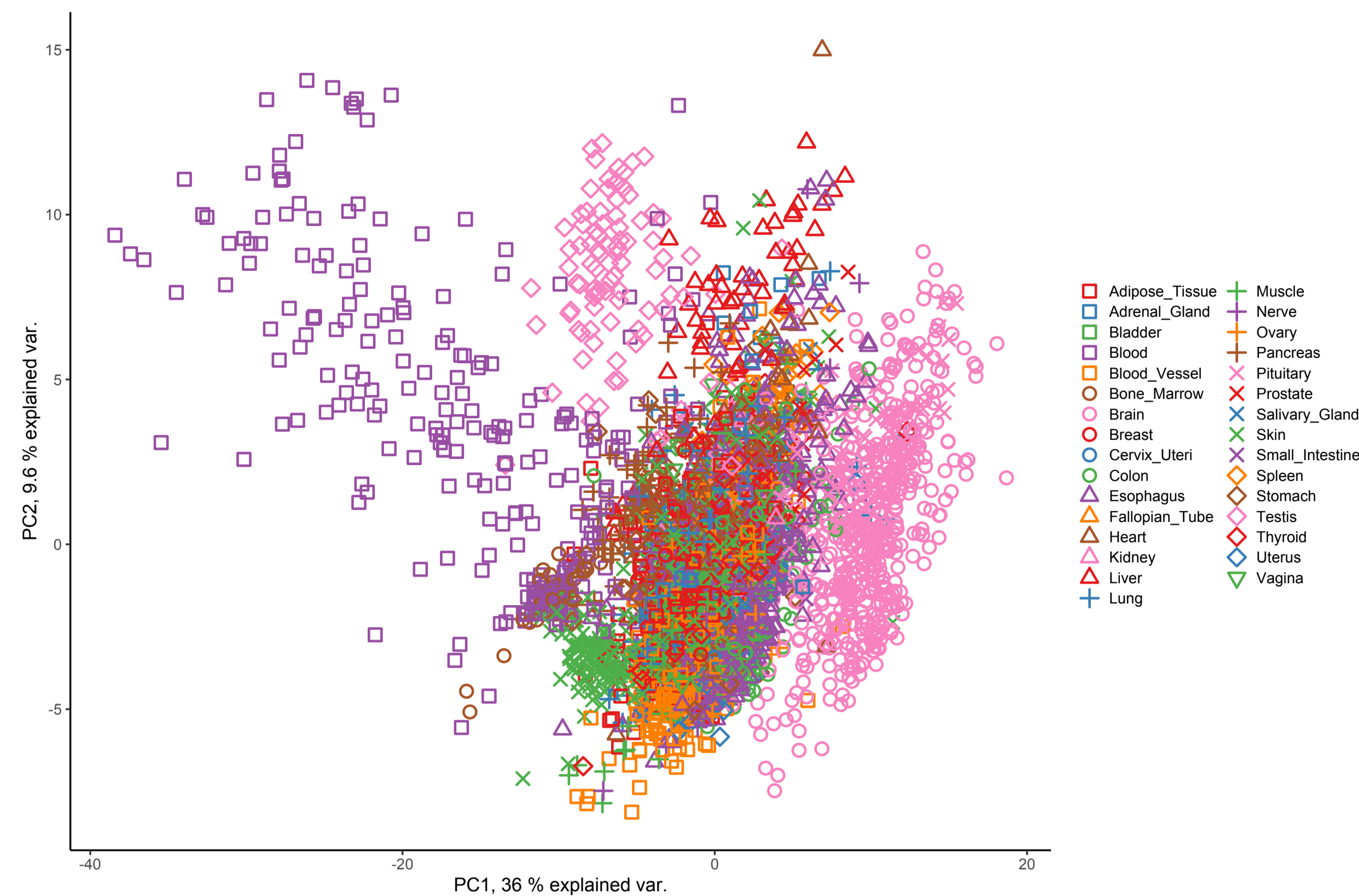

C

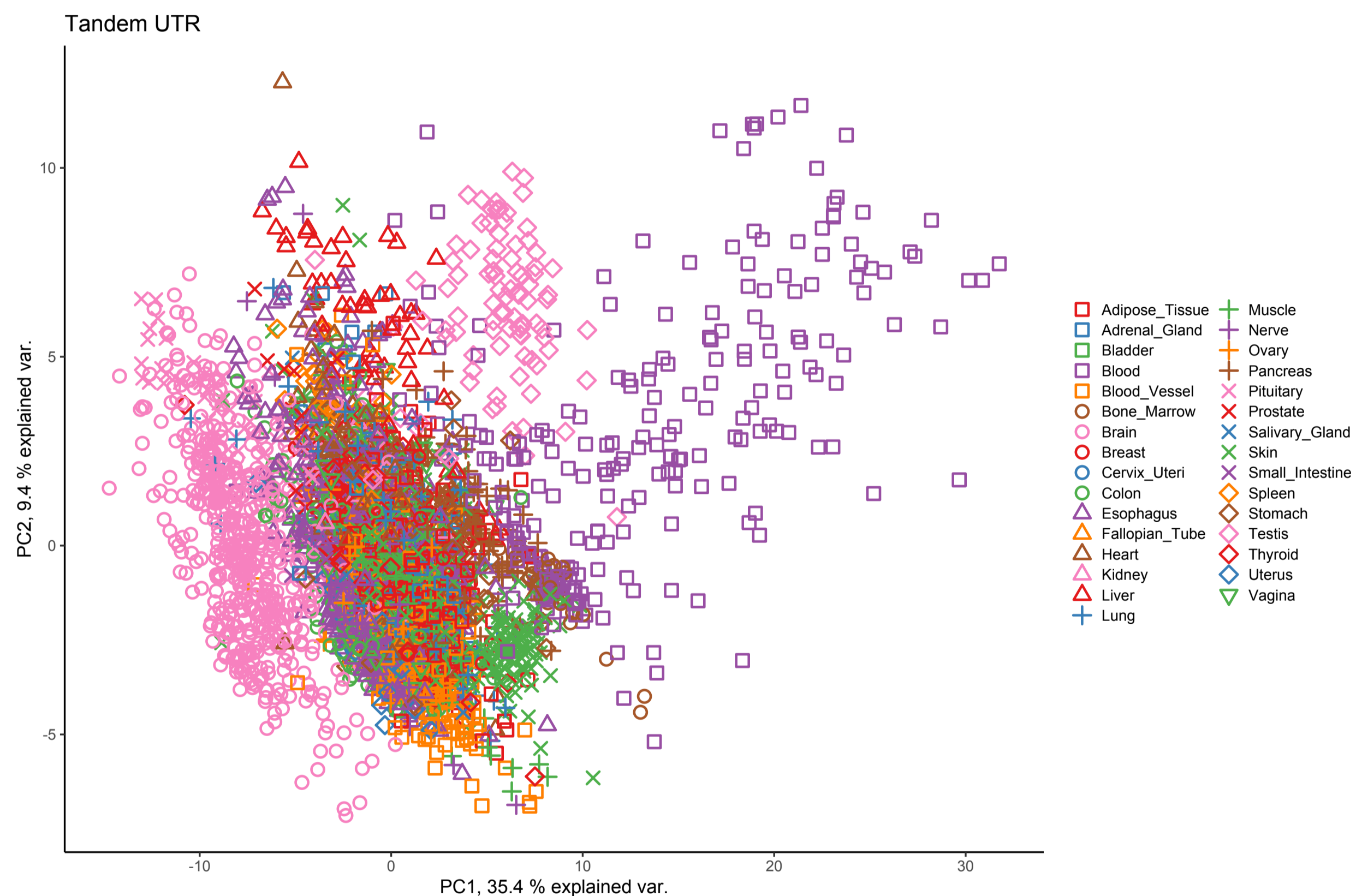

D

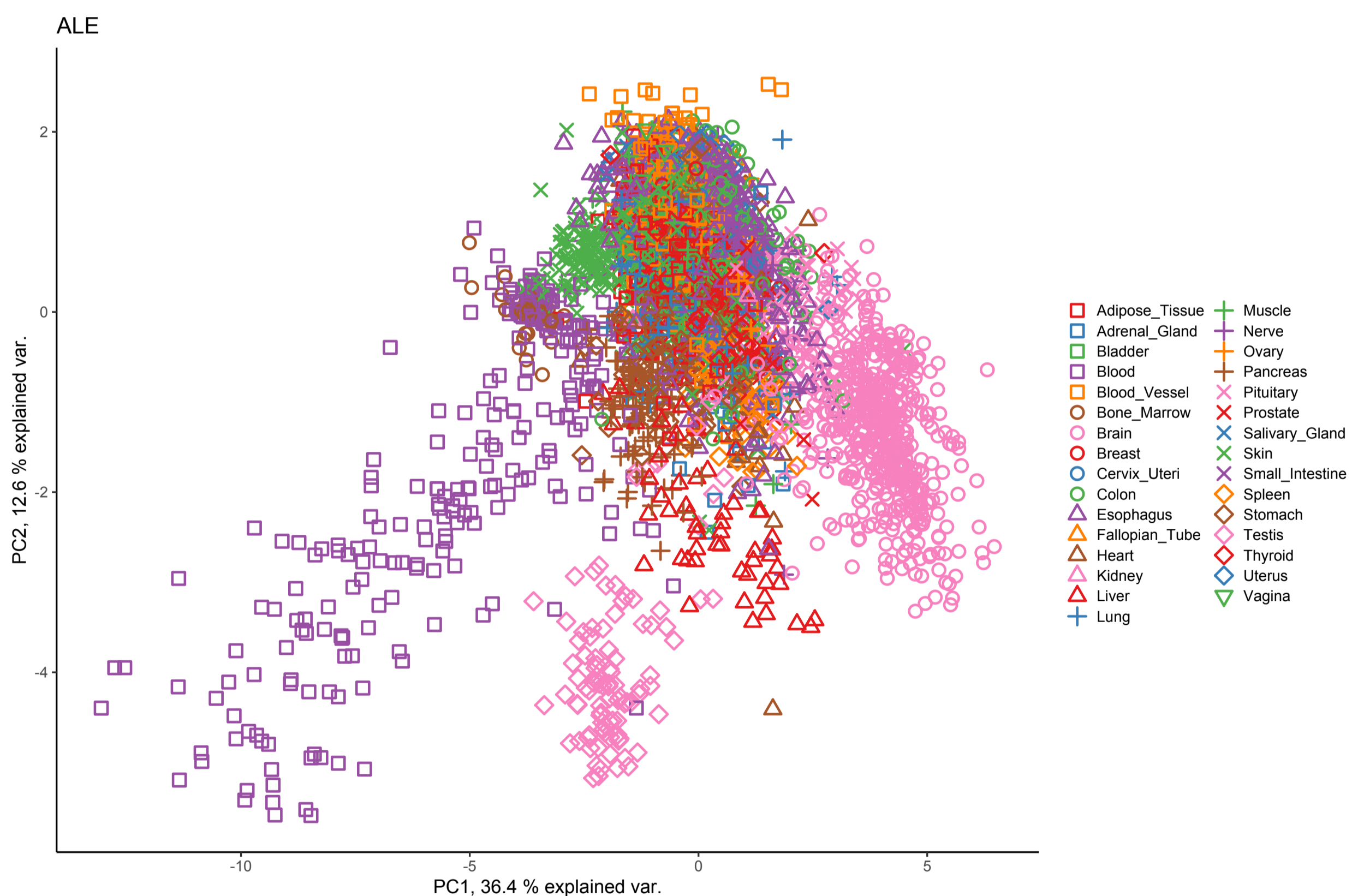

I

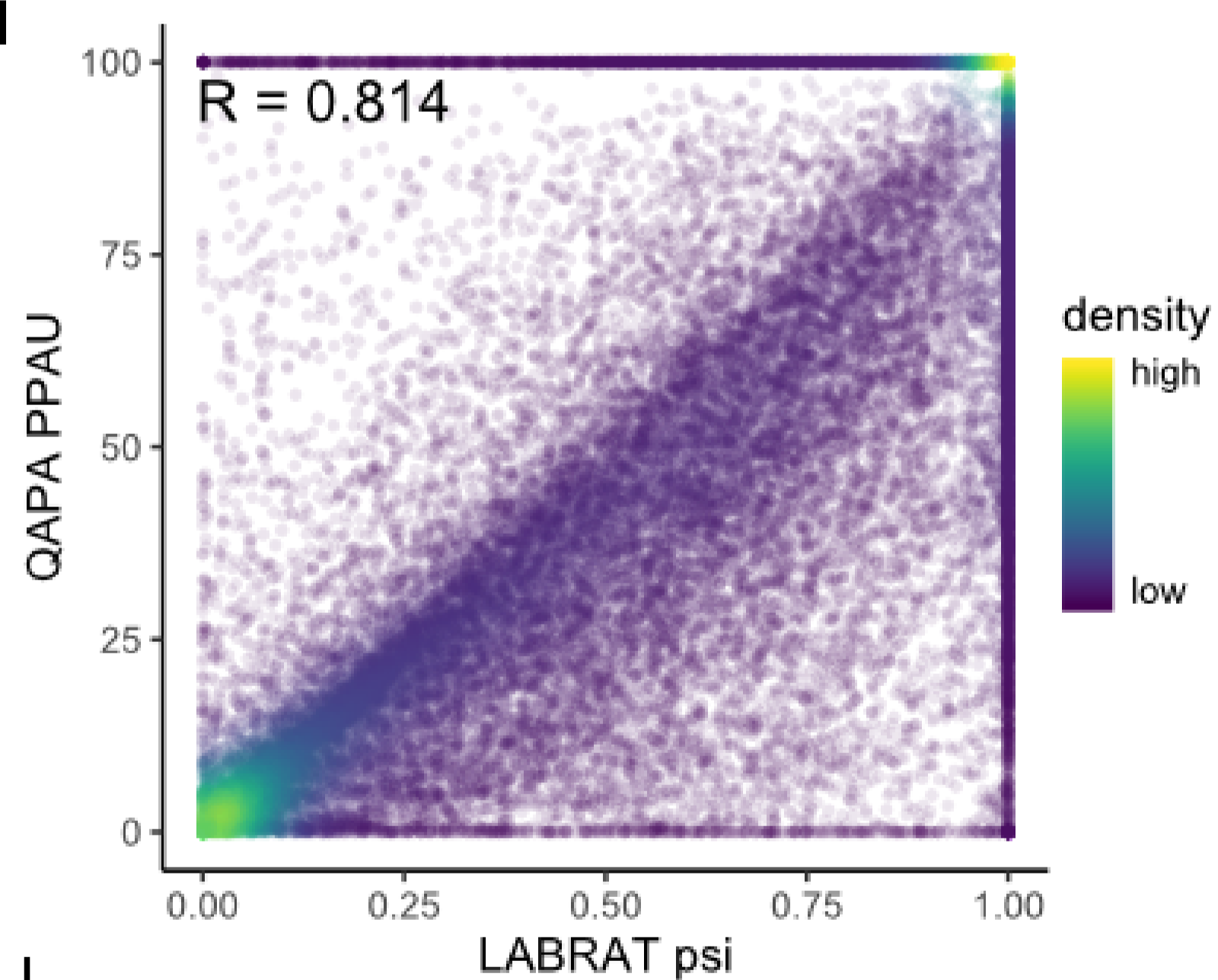

E

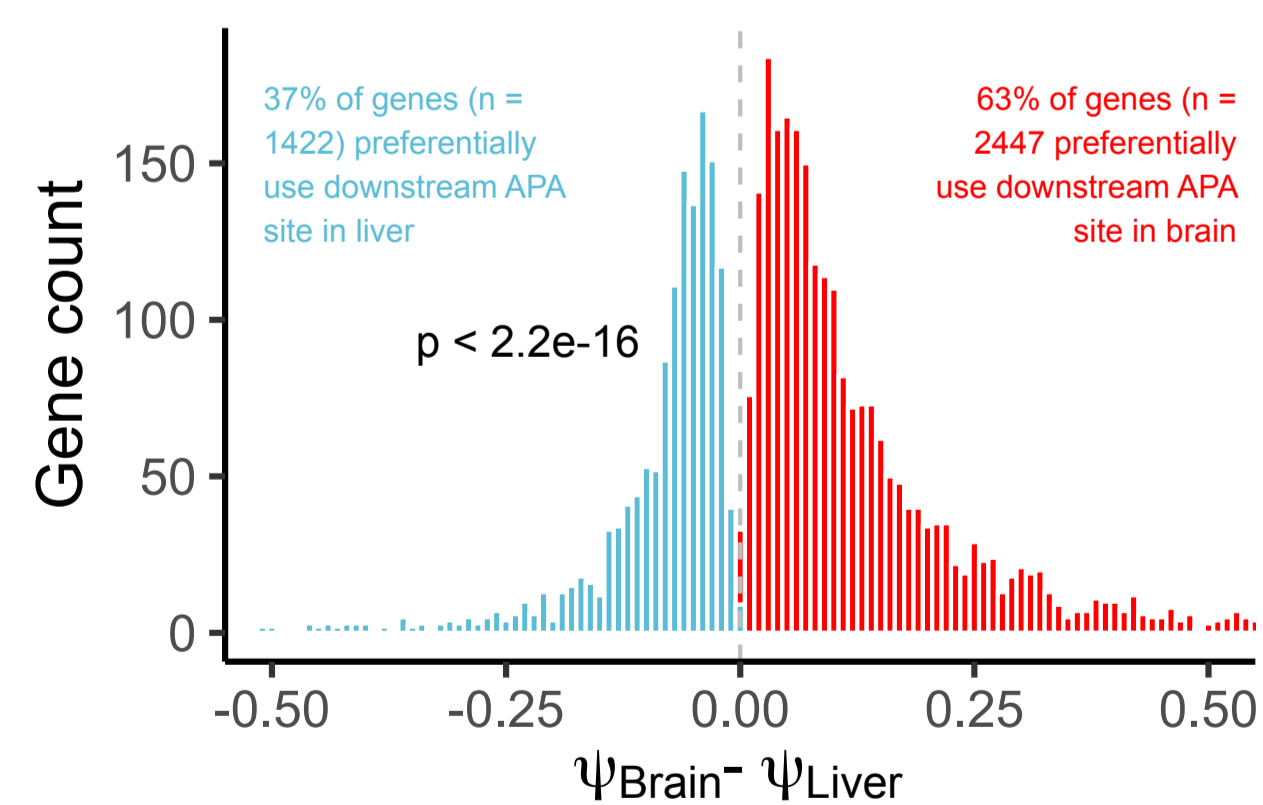

F

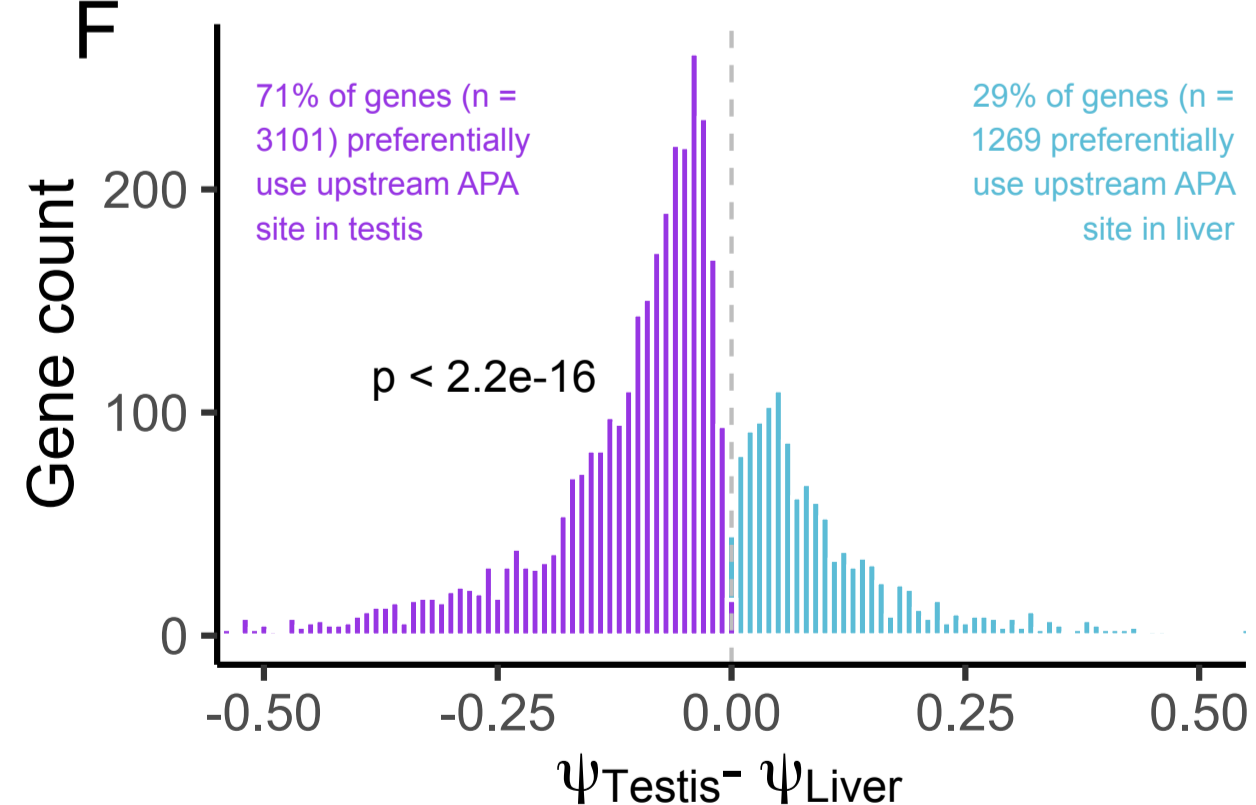

H

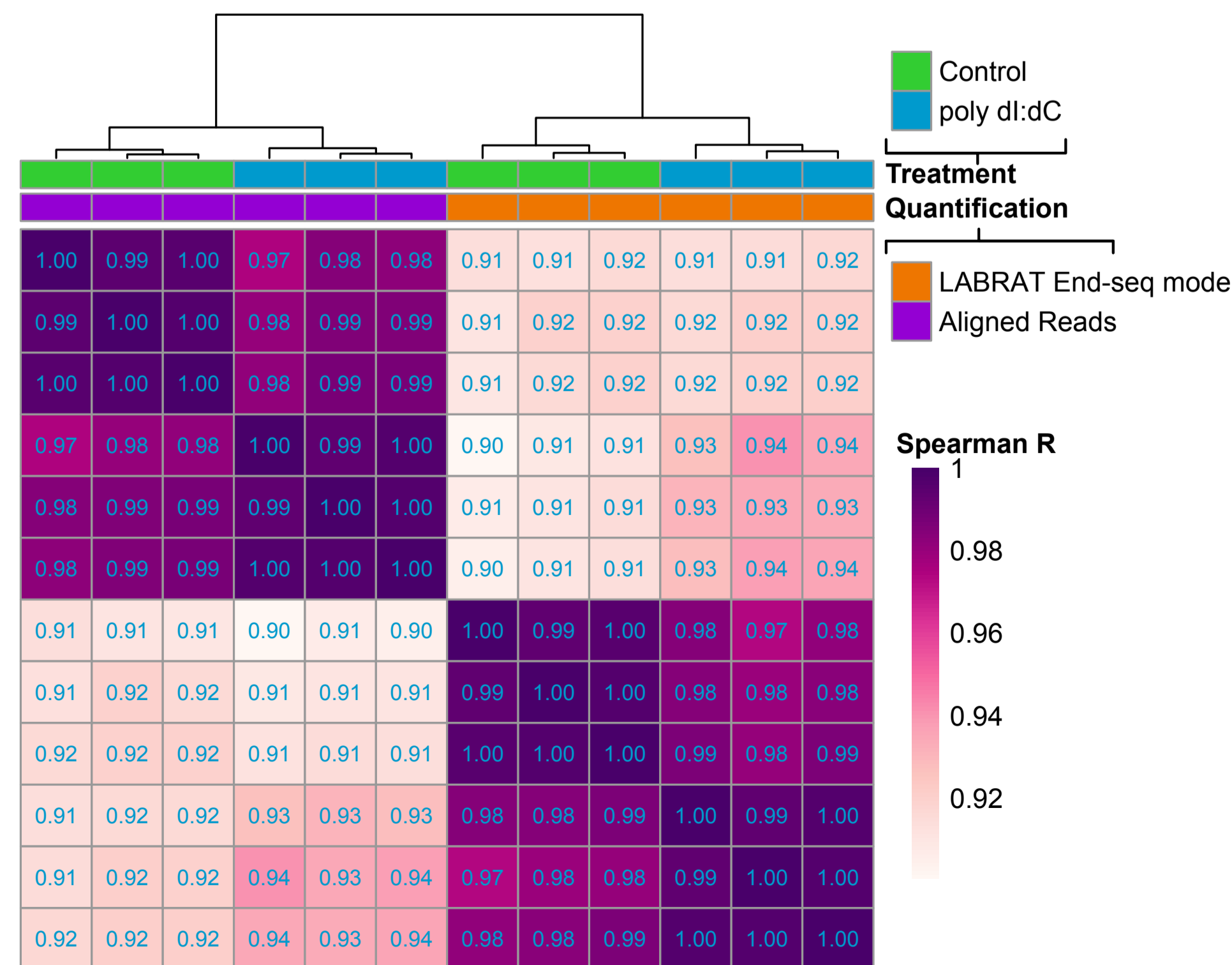

J

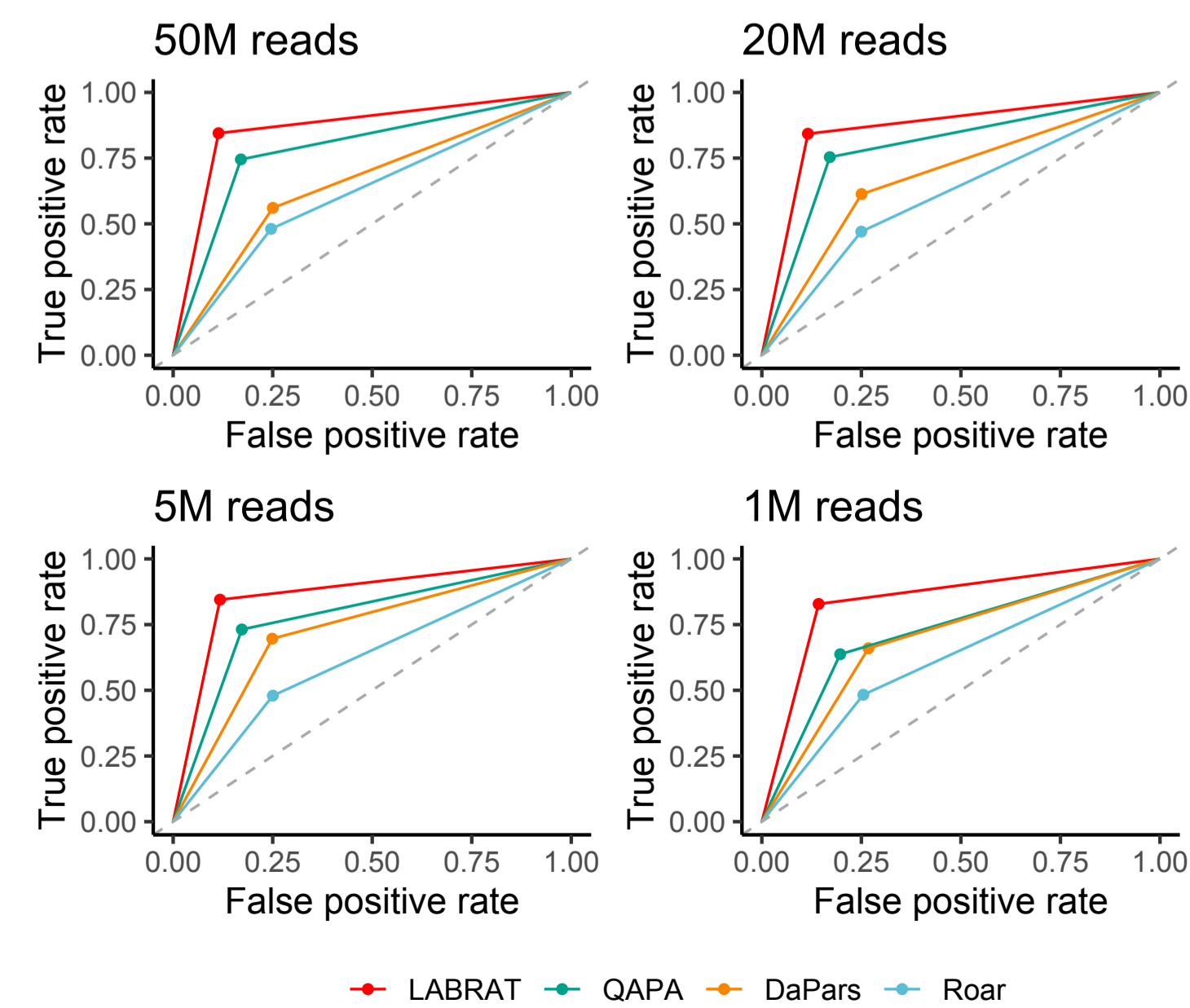

G

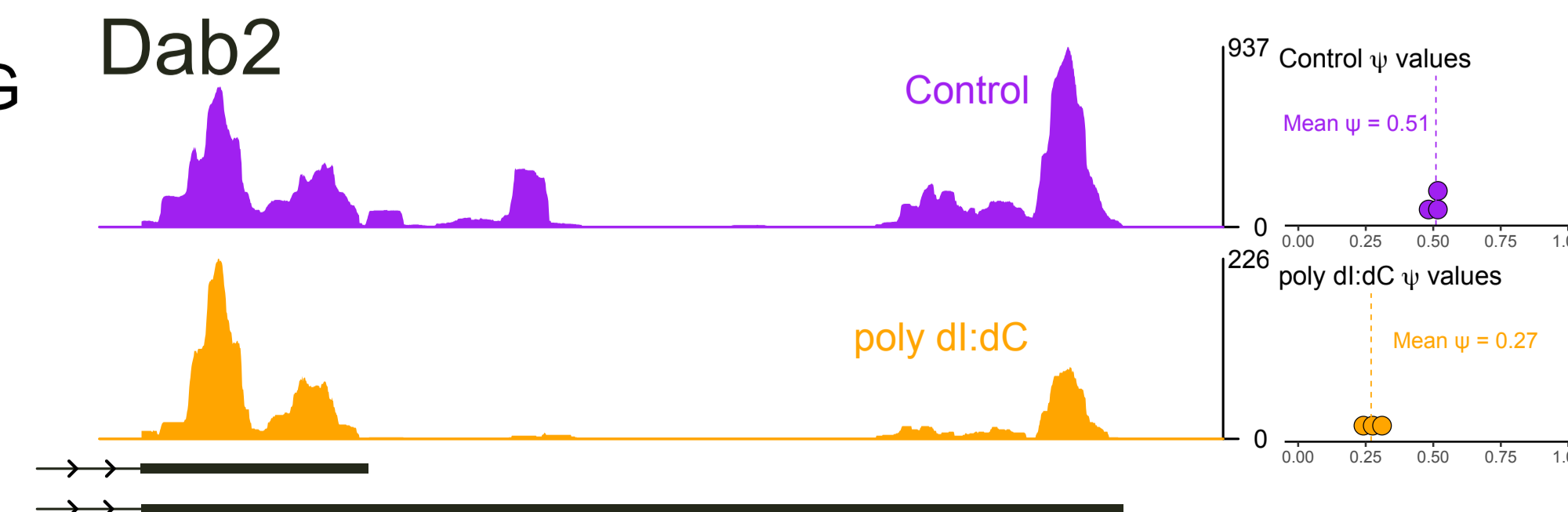

Supplement: Supplementary file 1 — Additional file 1: Figure S1. (A) Read coverage plot of Elavl1 in mouse brain and liver tissues. Dots represent ψ values of 8 replicates. (B) PCA analysis of ψ values calculated from human tissues. Data was produced as part of the GTEx project. (C) As in B, but only using genes that have a tandem UTR APA structure. (D) As in B, but using only genes that have an ALE APA structure. (E) Comparison of ψ values from human brain and liver samples. Delta ψ values for genes with FDR values less than 0.01 are plotted. (F) Comparison of ψ values from human testis and liver samples. Delta ψ values for genes with FDR values less than 0.01 are plotted. (G) Read coverage plot of Dab2 in control human PBMCs and those treated with poly dI:dC. RNA from these cells was profiled using 3′ end sequencing. Dots represent ψ values calculated in each of 3 replicates. (H) Comparison of ψ values from 3′ end sequencing data as calculated by LABRAT (orange) and by counting aligned reads (purple, see Methods) (I) Comparison of APA quantifications produced by LABRAT (ψ) and QAPA (PPAU). (J) Benchmarking of APA software performance at a range of sequence read depths. [file 12864_2021_7781_MOESM1_ESM.pdf]

# A

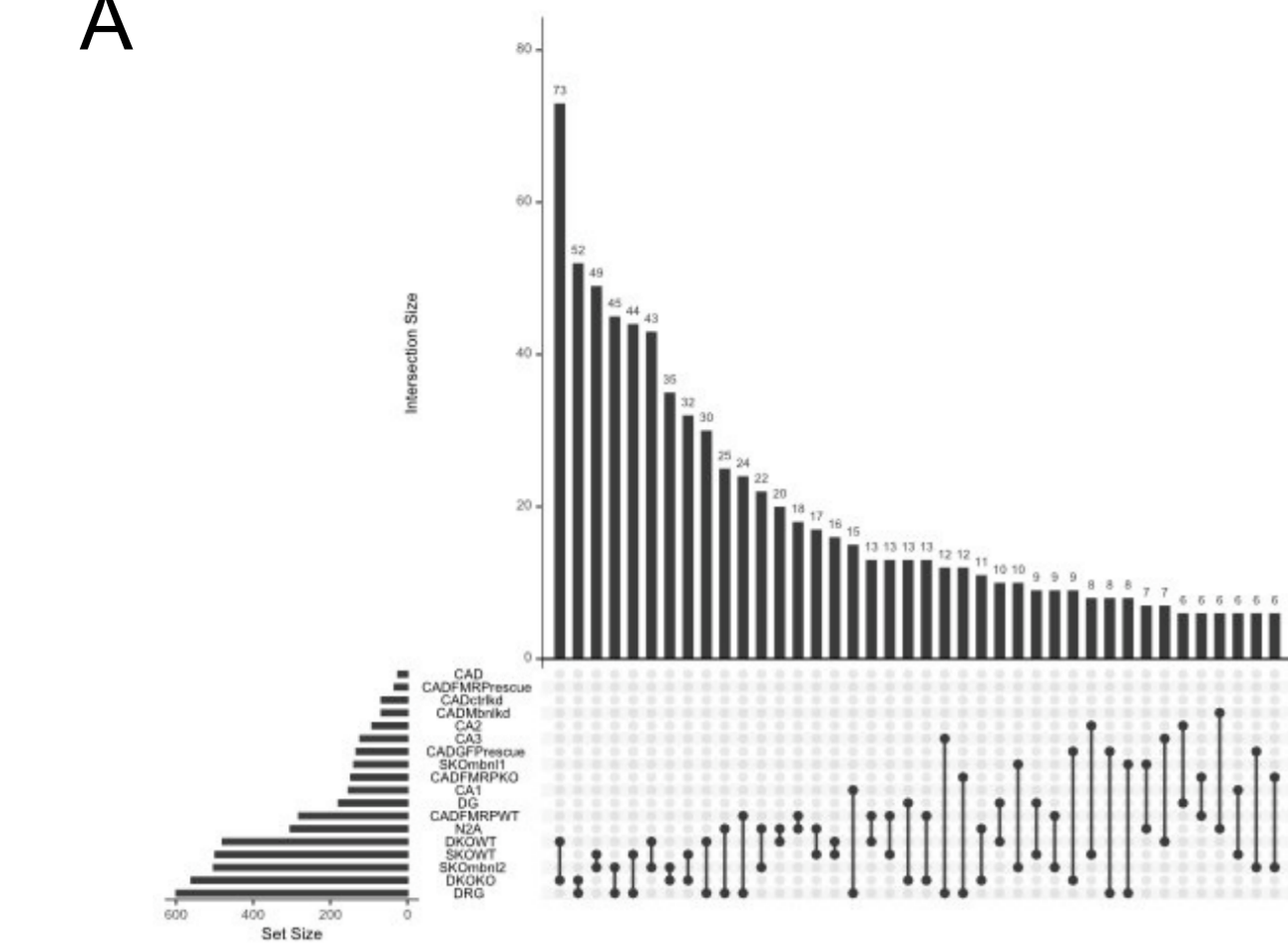

**B** Drosophila

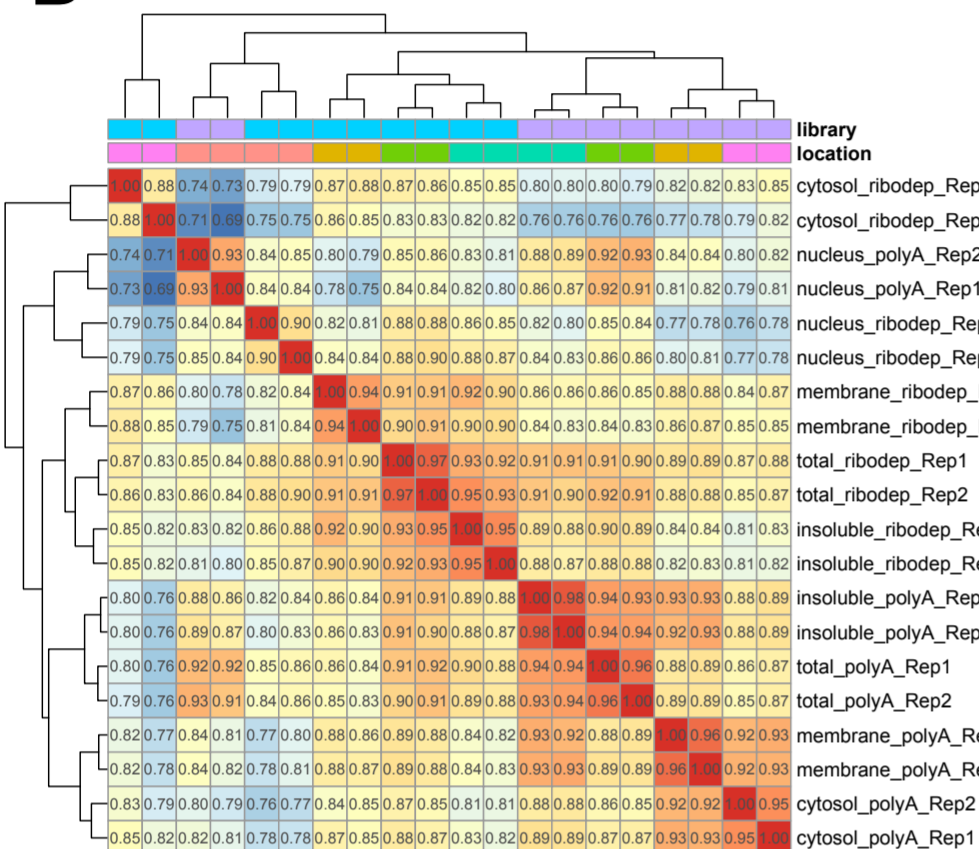

C HepG2

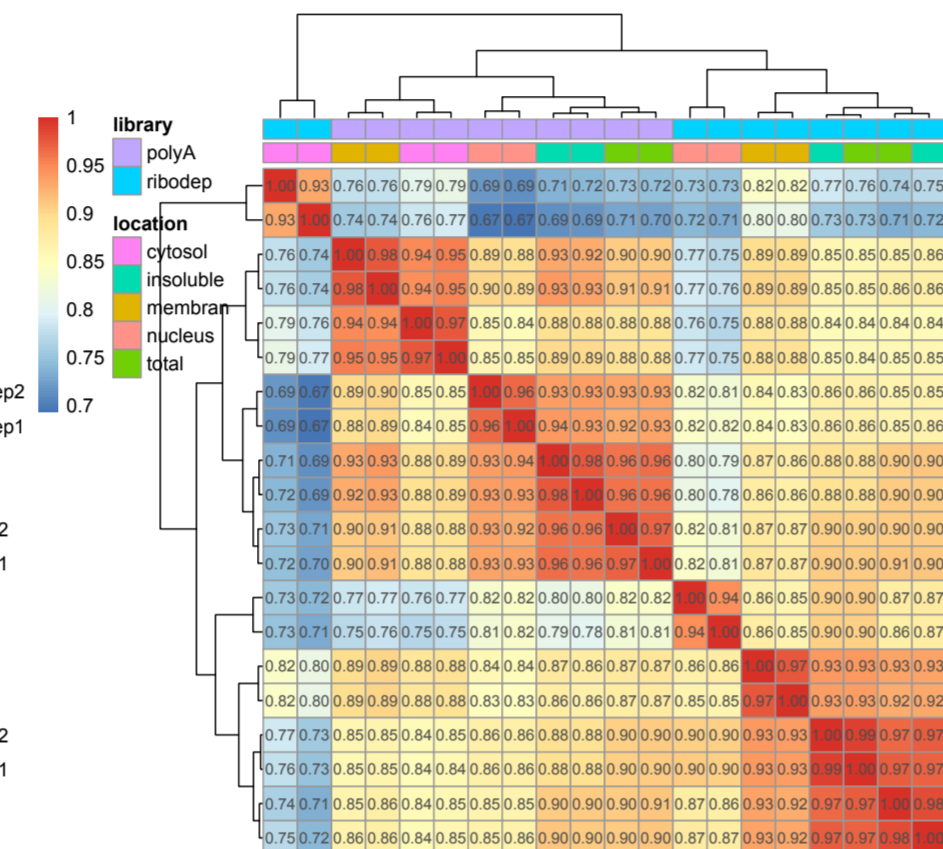

D K562

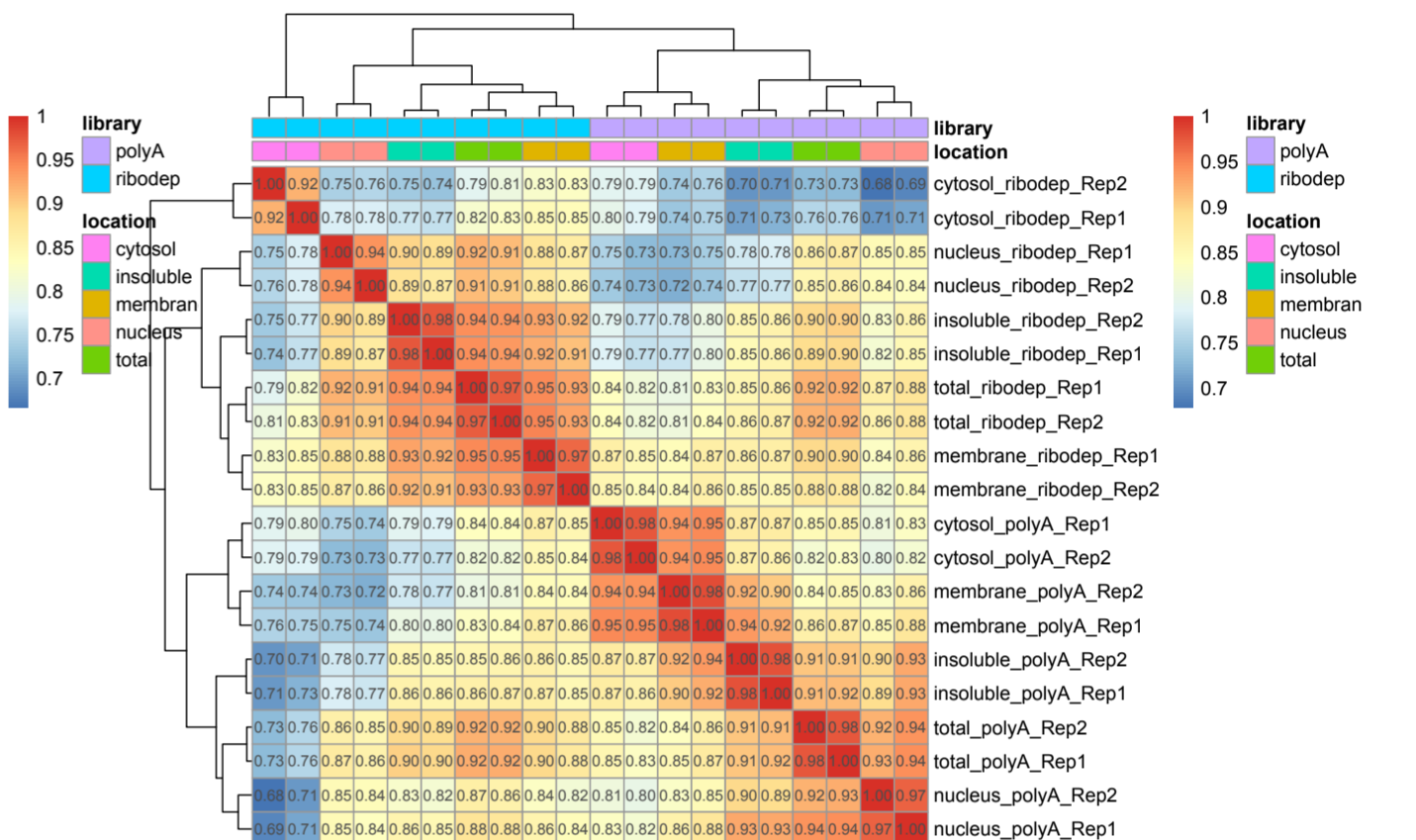

E

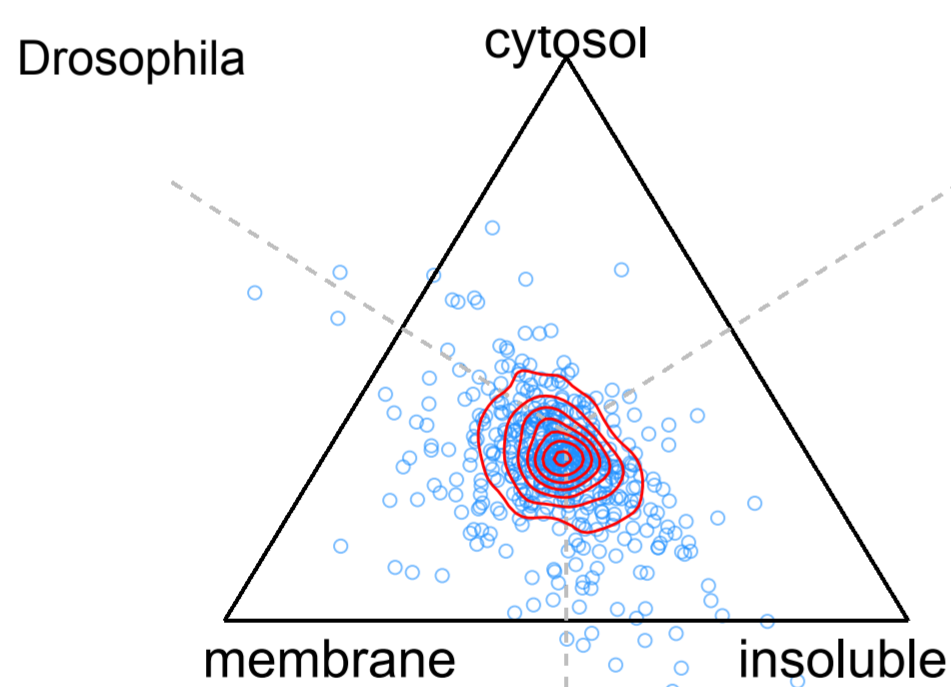

F

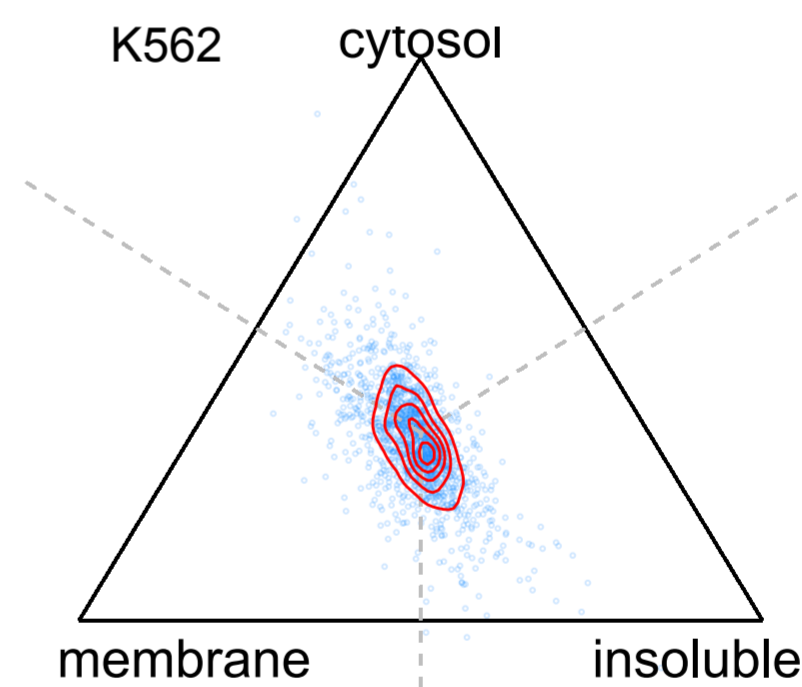

# G

# K562

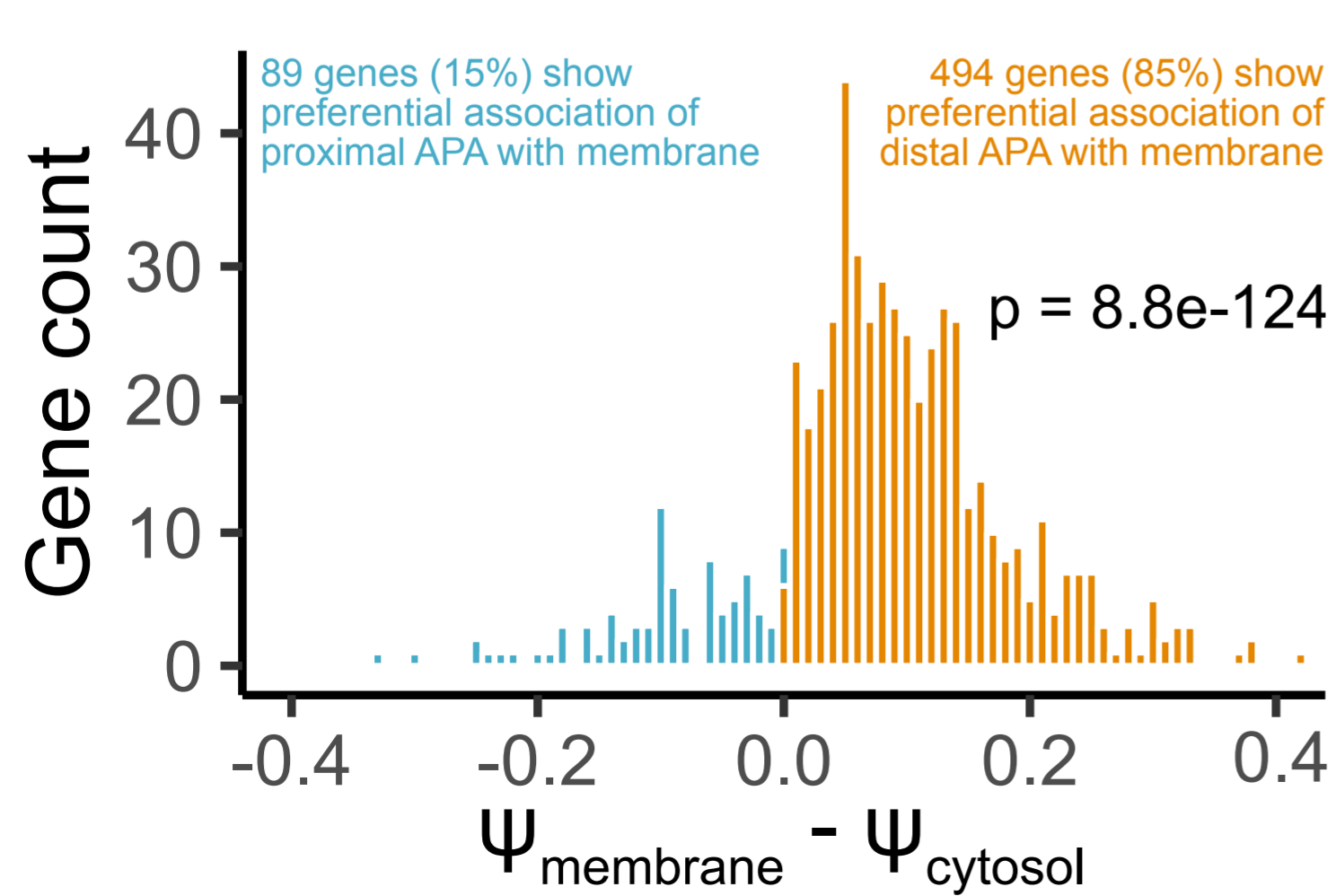

H

# ML-DmD17-c3

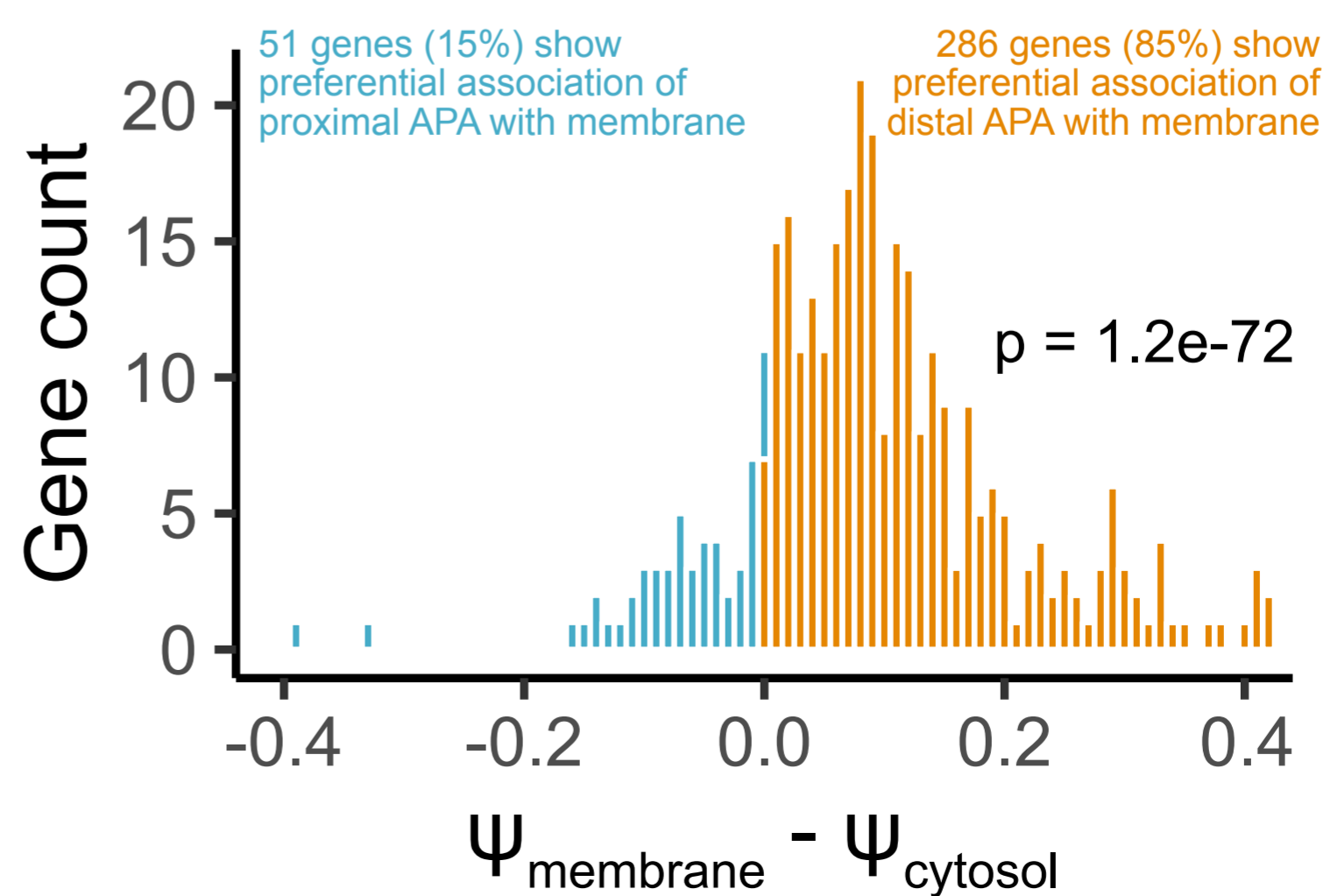

Supplement: Supplementary file 2 — Additional file 2: Figure S2. (A) Genes that repeatedly display differential APA isoform localization across repeated neuronal samples. Hierarchical clustering of ψ values from biochemically fractionated Drosophila DM-D17-C3 cells (B), HepG2 cells C), and K562 cells (D). (E-F) Simplex plots relating relative ψ values for genes between the cytosolic, membrane-associated, and insoluble fractions of DM-D17-C3 cells (E) and K562 cells (F). A dot that is equidistant from all three vertices had equal ψ values in each fraction while a dot that is closer to one vertex had a higher ψ value in that fraction relative to the other two fractions. (G-H) Comparison of ψ values in K562 (G) and DM-D17-C3 (H) cytosolic and membrane fractions for genes whose ψ value was significantly different between these compartments (FDR < 0.01). [file 12864_2021_7781_MOESM2_ESM.pdf]

**A****K562**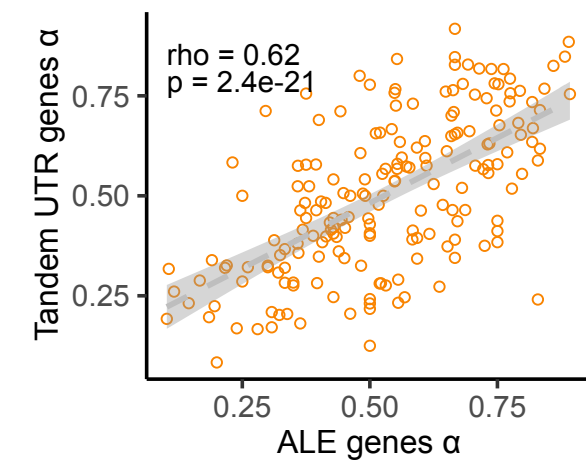**B HepG2**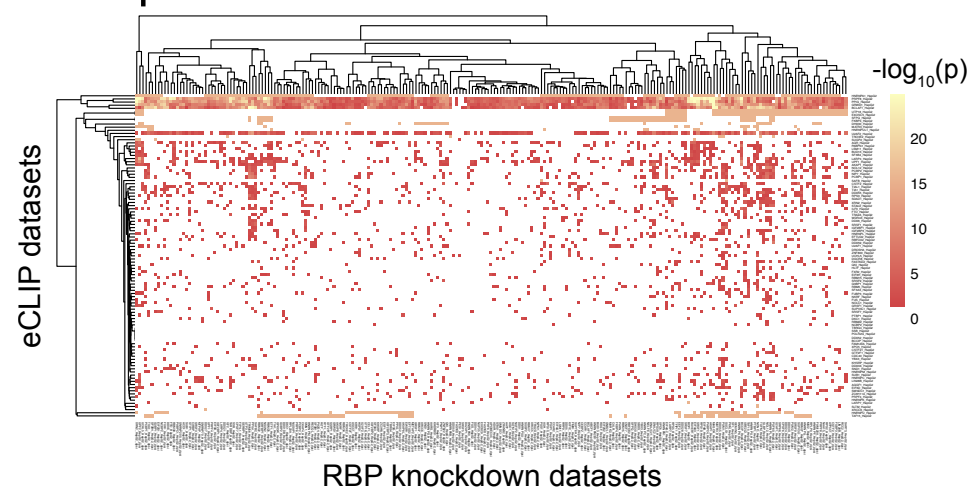**C****K562**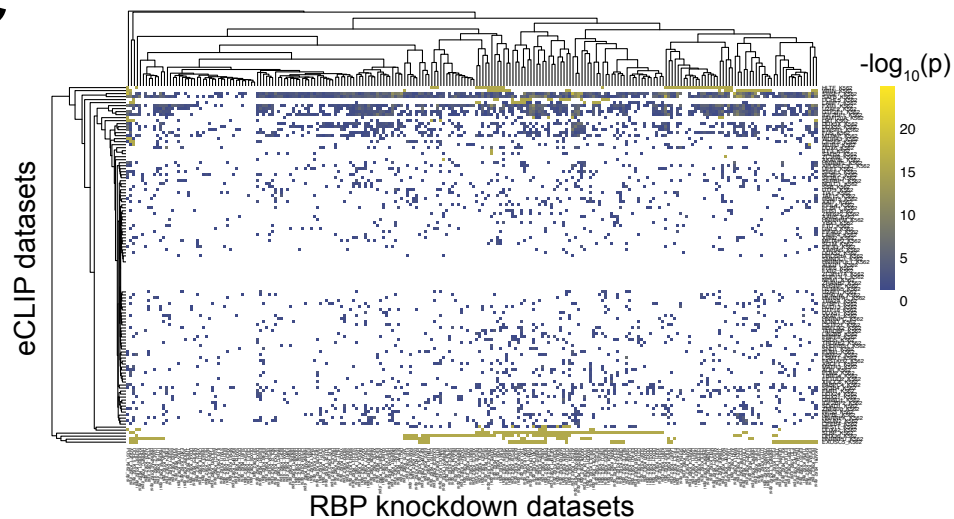**D****K562, 3' UTR binding events**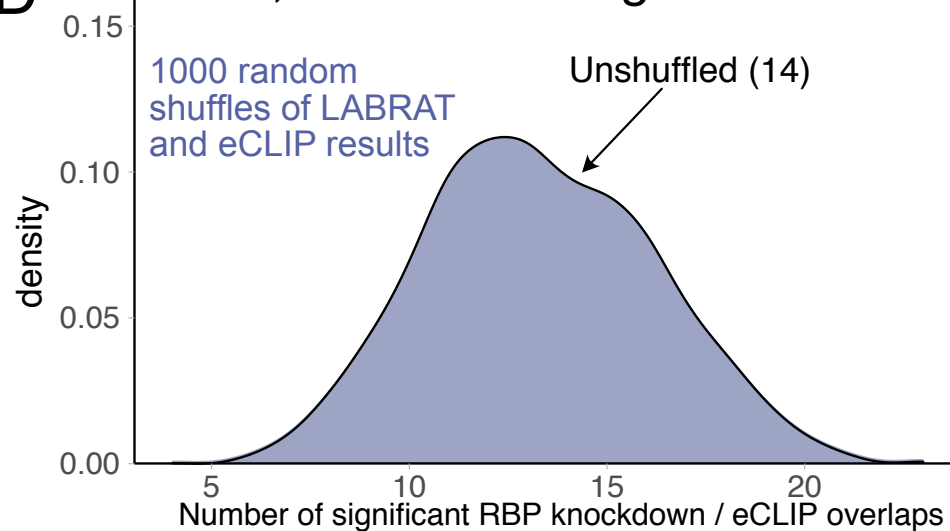**E****HepG2, whole gene binding events**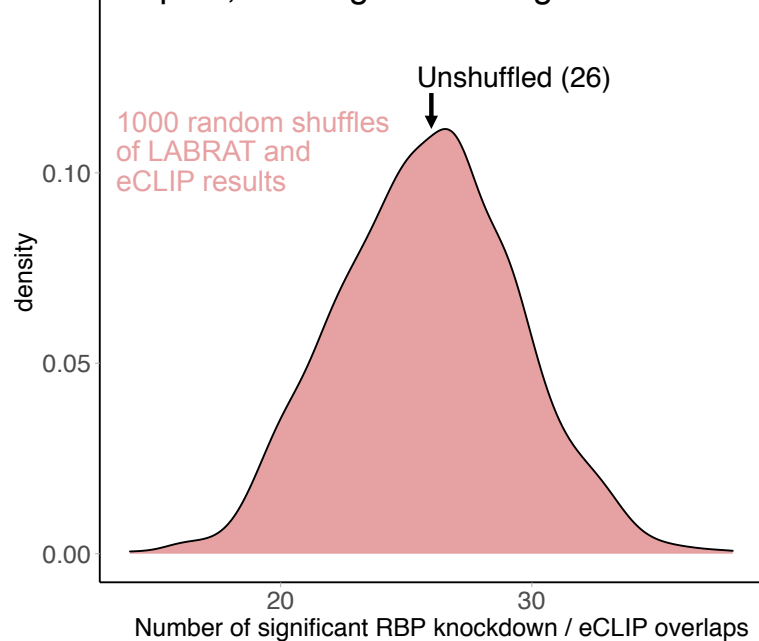**F****K562, whole gene binding events**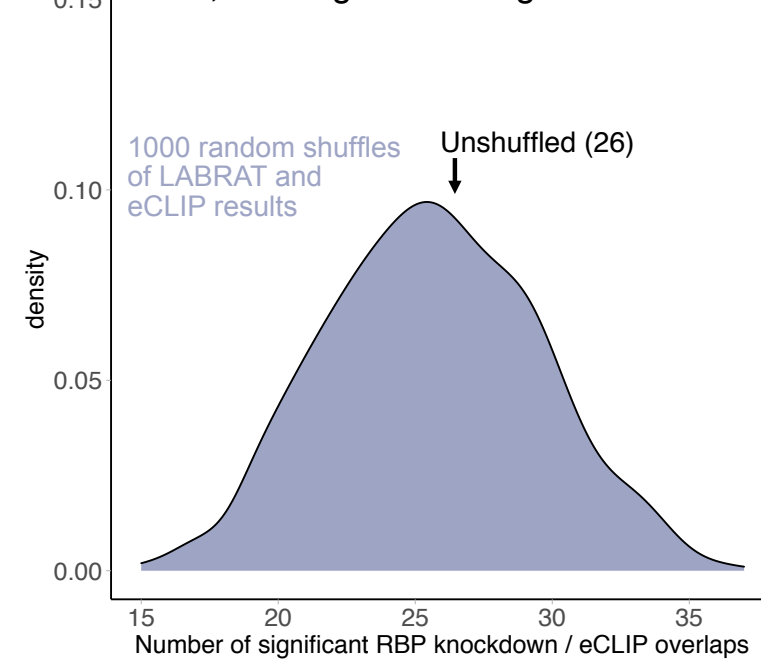

Supplement: Supplementary file 3 — Additional file 3: Figure S3. (A) α values for each RBP knockdown in K562 cells were calculated using tandem UTR and ALE genes independently. These were then plotted and correlated. Each dot in this plot represents one RBP knockdown experiment. (B) Binomial p values for overlaps between genes whose APA was sensitive to RBP knockdown and genes whose 3′ UTRs were bound by an RBP in eCLIP experiments. Data taken from ENCODE HepG2 experiments. (C) As in B, but using data from ENCODE K562 experiments. (D) As in Fig. 4E. Among 102 RBPs expressed in K562 cells, overlaps between the genes whose APA was sensitive to RBP knockdown and the genes whose 3′ UTRs were bound by the RBP in eCLIP experiments were calculated. The significance of this overlap was calculated using a binomial test. 14 RBPs bound the 3′ UTRs of their APA targets more often than expected (binomial p < 0.05). To assess whether this was more than the expected number of significant RBPs, relationships between RBPs and their lists of APA and eCLIP targets were shuffled 1000 times, and the analysis was repeated after each shuffle to create a null distribution (blue). (E, F) As in Figs. S3D and 4E, but instead of considering eCLIP binding events only in the 3′ UTRs of genes, eCLIP binding events throughout gene bodies were considered. [file 12864_2021_7781_MOESM3_ESM.pdf]

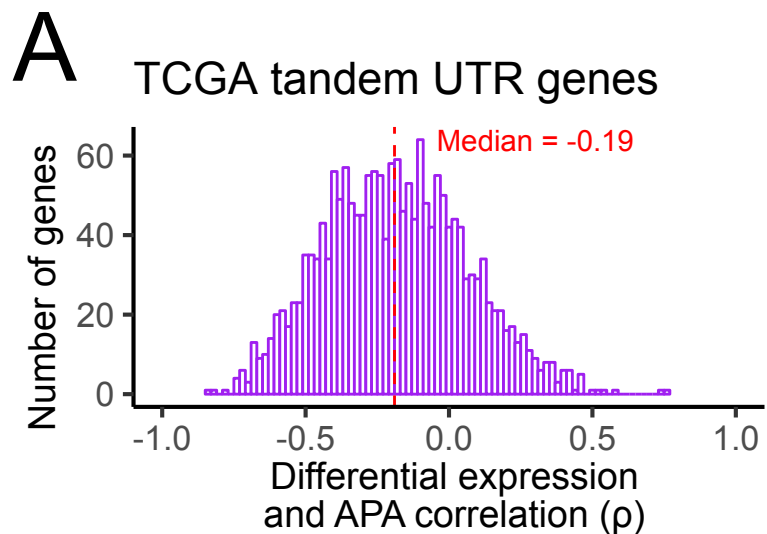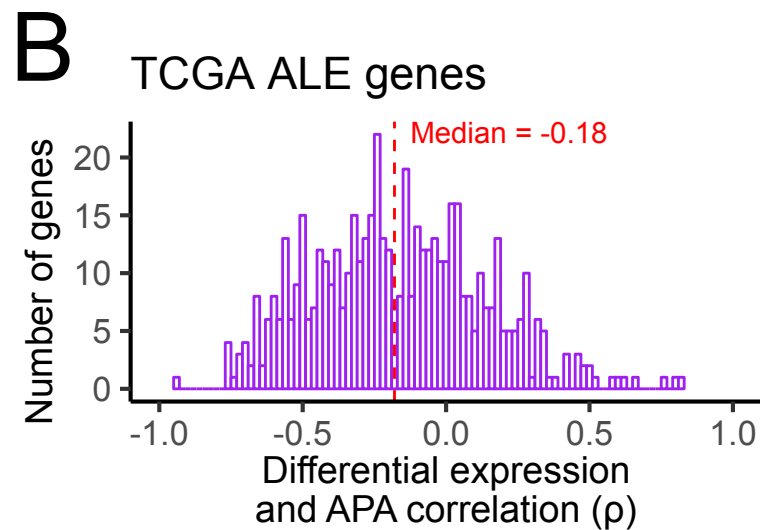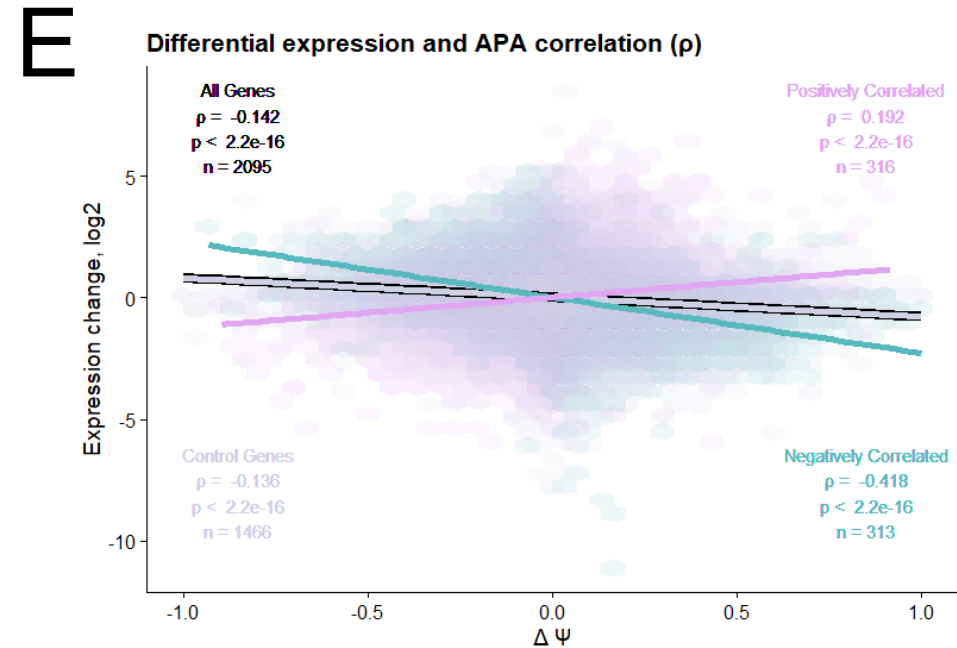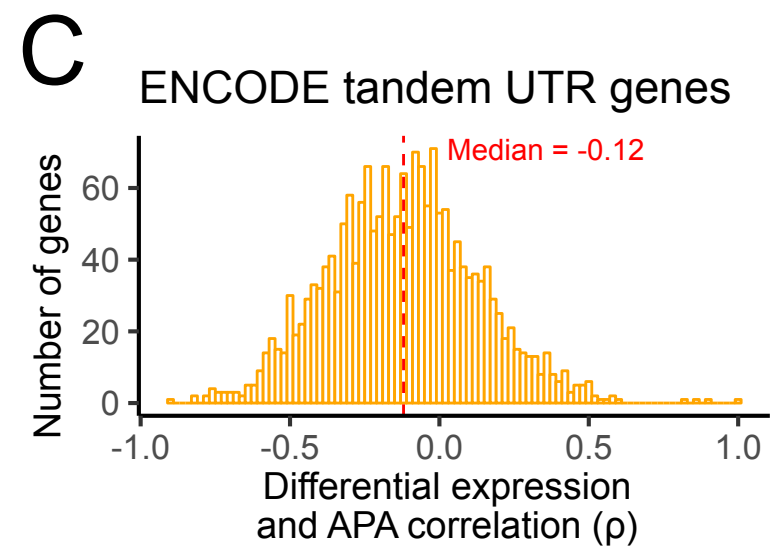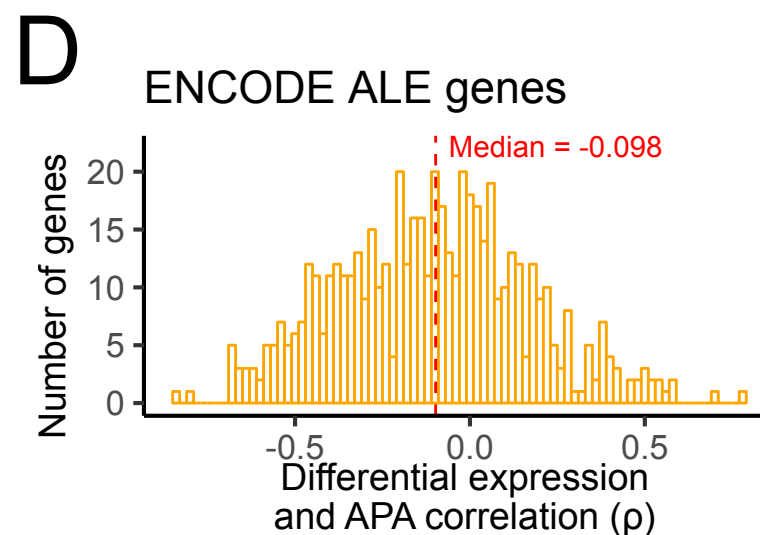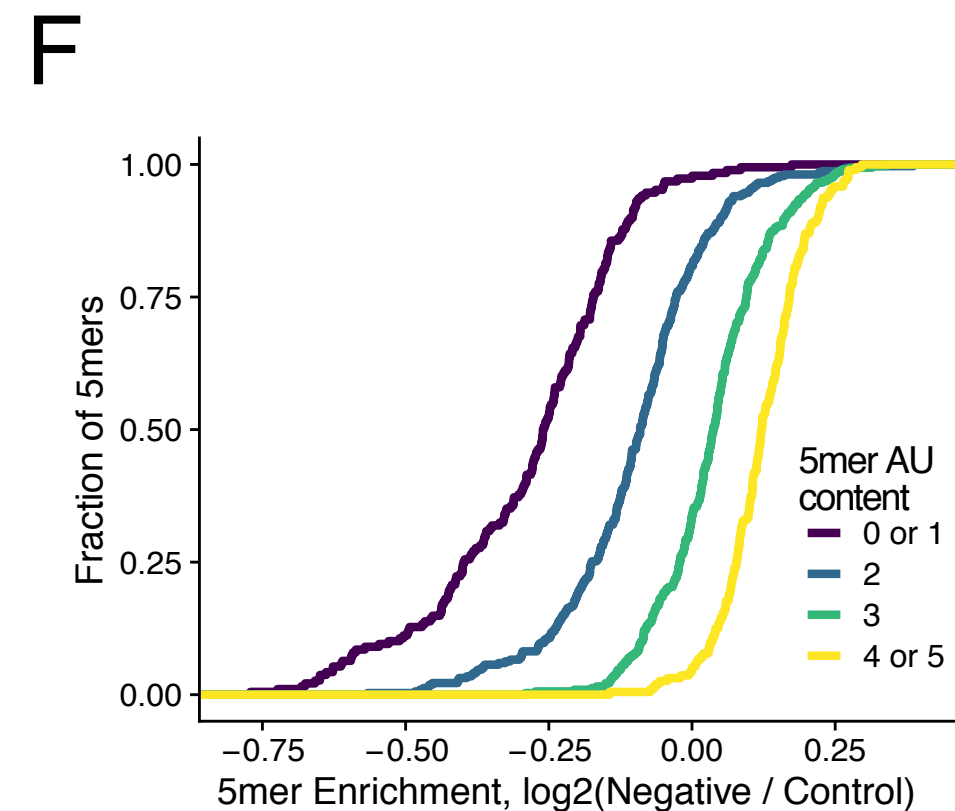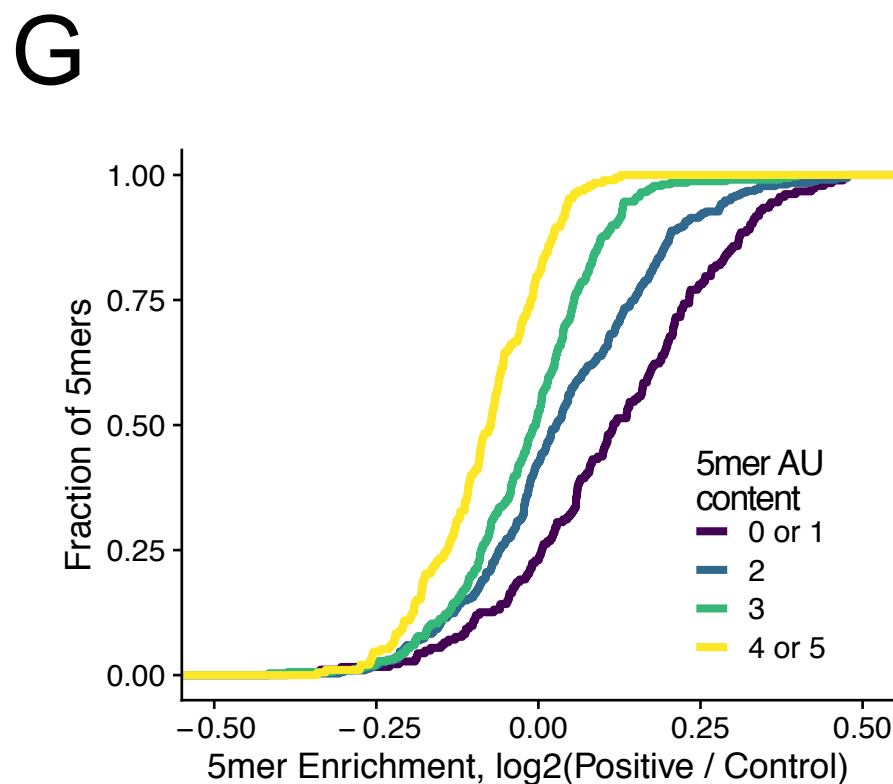

Supplement: Supplementary file 4 — Additional file 4: Figure S4. (A-B) Histogram of gene-wise correlations between changes in ψ and changes in gene expression (ρ) derived from TCGA tumor and matched normal samples for tandem UTR (A) genes and ALE (B) genes. (C-D) Histogram of gene-wise correlations between changes in ψ and changes in gene expression (ρ) derived from ENCODE RBP knockdown and control samples for tandem UTR (C) genes and ALE (D) genes. (E) Binned scatter plot comparing changes in ψ and changes in gene expression for genes with negative ρ values (blue), positive ρ values (purple) and control genes (gray). (F) Enrichment of 5mers in the distal UTRs of negatively correlated genes compared to the distal UTRs of control genes. (G) Enrichment of 5mers in the distal UTRs of positively correlated genes compared to the distal UTRs of control genes. [file 12864_2021_7781_MOESM4_ESM.pdf]
